# Clinical and Histological Healing after Maxillary Sinus Floor Elevation

**DOI:** 10.3390/dj10070134

**Published:** 2022-07-15

**Authors:** Daniele Botticelli

**Affiliations:** ARDEC Academy, Viale Giovanni Pascoli, 67-47923 Rimini, Italy; daniele.botticelli@gmail.com

**Keywords:** alloplastic, animal study, antrostomy, biomaterial, bone substitute, collagen membrane, dental implants, osteoconductivity, Schneiderian membrane, xenograft

## Abstract

Sinus floor elevation is a surgical procedure that allows for the insertion of the implant in the posterior region of the maxilla in case of insufficient volume of native bone. Several studies have reported a high success rate, and this has helped to spread this surgical procedure around the world. The subject has been extensively studied and this could lead researchers to think that no new scientific evidence can be provided. However, new ideas and discoveries show that research never reaches a conclusion, and that new information can be added all the time. This collection aimed to provide new evidence that could be added to daily clinical work and to provide new ideas for future research.

## 1. Introduction

The posterior segment of the maxilla often present insufficient bone volume for implant installation. Sinus floor elevation, applying a lateral access, is considered a reliable surgical procedure to increase bone volume in that region [1,2,3]. However, before performing such treatment it is very important to accurately assess the anatomical conditions of the sinus on a cone beam computed tomography (CBCT) [4,5] to avoid technical problems and complications during surgery, and short and long term post-operative problems [6]. The anatomical conditions might complicate the surgical approach, such as the presence of septa [7,8,9], or of an acute palatal-nasal recess [4,10]. The presence of cysts within the sinus cavity will force the clinician to evaluate the possible solutions [11,12], while the position of the superior posterior alveolar artery might interfere with the surgical procedures [13].

During surgery, the most frequent complications are bleeding and sinus mucosa perforations [6]. Small perforations may not need any action [14], while larger perforations might be closed with sutures [15,16,17] or fibrin glue [15,16,18], or protected with a collagen membrane [19,20,21,22,23,24].

The most common complication after surgery is the bleeding within the elevated space and the edema involving the sinus mucosa. These events result in a transient thickening of the sinus mucosa [19,20,25,26,27,28,29,30] that might extend towards the ostium, decreasing its diameter, and compromising the patency [31]. Other complications might be wound dehiscence [32], extrusion of biomaterial inside the antral cavity [33,34], and sinusitis [32,33,34,35].

Despite the large amount of literature on sinus floor elevation, further evidence may be useful for increasing the knowledge in the field. All experimental and clinical papers included in this collection have addressed various aspects related to sinus floor elevation. The contribution of several experienced clinicians and researchers has enabled this collection of articles to be generated.

## 2. A Synopsis of the Special-Editions Papers

The present collection was made possible thanks to the collaboration between ARDEC Academy, Rimini (Italy), and highly reputable Universities and Clinical institutes located in Austria, Brazil, Colombia, Israel, Italy, Japan, Spain, Russia, and USA. This collection on clinical and histological healing after sinus floor elevation contains 10 articles, of which 5 were experimental studies in rabbits and 5 addressed clinical issues.

### 2.1. Experimental Studies

In the first experimental study included in the present collection, the healing in the maxillary sinus after the placement of deproteinized bovine bone matrix was evaluated [36]. Granules of different size were used, 0.125 mm or 1–2 mm and three periods of healing were analyzed, i.e., 2, 4, and 8 weeks, six rabbits for each period. Bone apposition within and onto the biomaterial was evaluated histologically in various zones of the sinuses. It was reported that in the first stages of healing, the granules were covered by dense and loose connective tissues. The dense tissue was substituted over time with new bone while the loose tissue took on similar characteristics to marrow spaces. No differences were found in bone formation and osteoconductivity between the two groups in any period and regions evaluated.

In the second experiment, granules or paste composed of beta-tricalcium phosphate and hydroxyapatite were used to fill the elevated subantral spaces [37]. The healing was evaluated in various regions of the elevated region after 2 and 10 weeks, ten rabbits per period. Higher percentages of new bone were found at the sites grafted with granules than those with paste. However, the gel contained in the paste was not resorbed completely after 10 weeks. It was concluded that bone formation was faster at the granules compared to the paste sites. However, longer periods should be evaluated to allow a more complete resorption of the gel fraction of the paste that might allow further bone apposition.

In both studies all biopsies were analyzed in microCT. Only volumetric data were reported due to the difficulty in determining the grayscale (threshold) that accurately differentiated new bone from the remaining graft, as already reported by other study from the same group of research [38,39].

The third experiment was conducted to provide further evidence to the conflicting results reported on the influence of a collagen membrane placed to protect the access window after sinus floor elevation [40]. After the elevation of the sinus mucosa, a collagenated cortico-cancellous porcine bone was grafted into the elevated space bilaterally. The collagen membrane was placed to protect only one of the two osteotomies. Three periods of healing were analyzed, i.e., 2, 4, and 8 weeks, using eight rabbits per period. No differences were found between groups so that it was concluded that the placement of a collagen membrane on the access window did not influence the amount of new bone after sinus grafting. This corroborated the results from other studies performed by the same research group [41,42].

The fourth study [43] was designed to offer one more evaluation of the thinning and perforations of the sinus mucosa in contact with biomaterial granules observed in previous studies from the same group [44,45]. In the present study, two periods of healing were evaluated, 2 and 10 weeks, ten rabbits for each period. Two different types of deproteinized bovine bone in granules, one processed at low temperature (low-T group), and the other at high temperature (high-T group), were used. Both biomaterials presented hundreds of thinned mucosa sites in contact to the graft granules, number that increased between 2 and 10 weeks. Perforations of the sinus mucosa, with extrusion of the biomaterial into the sinus, was found already after 2 weeks, reaching the number of 19 in the low-T group, and 14 in the high-T group after 10 weeks. No statistically significant differences were found between groups, even though a trend of higher number of thinning mucosa and perforation was found at the low-T compared to the high-T group.

The fifth experiment was performed to evaluate the effect on osseointegration of the treatment of the implant surface with argon plasma but also to assess a possible rate of perforation of the sinus mucosa [46]. The assumptions were based the former on a study of dogs that showed better osseointegration at implants that received the argon plasma treatment [47]. The latter was based on two studies, one on osteoconductivity in sinuses elevated with deproteinized bovine bone matrix in rabbit sinuses [48], and another that showed thinning and perforations of the sinus mucosa in contact with threads and apex of the implants [45]. In the present experiment [46], 16 rabbits were included in the experiment. The sinus mucosa was elevated bilaterally after the preparation of an access window and implants were installed immediately without grafting the subantral spaces. After 8 weeks of healing, no statistically significant differences were found between plasma and control sites in bone apposition, as well as in thinned sties and perforation. A high number of perforations were observed, 12 at the apexes and 15 at the threads in the plasma group, and 12 at the apexes and 18 at the threads in the control group. Moreover, in both groups, more than 50 sites presented a thinned mucosa <40 µm (mean thickness of 14–15 µm) while the pristine sinus mucosa was about 80 µm.

It has to be considered that, when the data from animal studies are inferred to humans, some considerations have to be kept in mind, such as the smaller dimensions of the sinus and of the sinus mucosa in rabbits [49,50], and the faster healing rate in animals compared humans [51].

### 2.2. Clinical Studies

The first clinical article included in the present collection was a retrospective study on the anatomical parameters that might influence the thickening of the sinus mucosa and the dimension of the subantral space after sinus floor elevation [52]. For the evaluation, the axis (*x*-axis) drawn at the base of the nose was used as reference, as suggested in various studies published by the same research group [4,19,20,29,30,31]. The base of the nose corresponds to about the level of the palatal-nasal recess that is an important reference for the surgeon. In the region delimited by the *x*-axis, the sinus floor and the lateral and medial sinus walls, the implants are to be installed. The distance between the *x*-axis and the sinus floor (X-F) and the residual bone crest are two important factors that influence the amount of sinus elevation necessary for implant installation. A weak significant positive correlation was observed between height gain vs. sinus height of interest (XF), vs. the distance between the base of the access window and the sinus floor, and vs. the sinus floor angle. The post-surgical edema was influenced by the initial mucosa thickness and the xenograft used.

The second clinical study evaluated the effect on implant osseointegration on a collagen membrane subjacent the sinus mucosa [53]. Twenty patients participated in this randomized clinical trial. Ten patients received a collagen membrane and ten did not. After 6 months from sinus lifting, a mini-implant was placed transcrestally and retrieved after 3 months. It was concluded that the use of a collagen membrane subjacent the sinus mucosa did not affect osteointegration confirming what it was observed in experimental studies in sheep [54,55] and in rabbits [49].

The problem of the removal of antral pseudocysts was addressed by the third clinical study [56]. In total, 86 pseudocysts were removed from 52 sinuses of 46 patients. The diagnosis was confirmed histologically for all lesions. After 4 months of healing, the sinus floor elevation was performed. Cone beam computed tomographies (CBCT) were taken at various periods of healing. It was suggested to apply a two-stage approach to allow the evaluation of the healing of the lesion and the histological assessment.

The presence of septa might complicate the sinus lifting procedure and increase the rate of perforations. In the fourth paper [57], the thickening of the mucosa and the involvement of the ostium after sinus lifting were analyzed in 15 sinuses with septa (test) and 15 without (control). CBCTs taken before the surgery and after 1 week and 9 months were taken. Four perforations occurred at the septa sinuses while none were observed at the control group. After 1 week of healing, the thickness of the sinus mucosa increased more at the control (7.1 mm) than at the test (5.7 mm) sites. The ostium was more involved in the control compared to the test group. Five ostia in the control and three in the test groups appeared to be obstructed. However, after 9 months from surgery, sinus mucosa and ostium regressed to normality.

The last clinical paper is a systematic review dealing with the alteration of voice and speech after sinus floor elevation (SFE) or maxillary functional endoscopy surgery (FESS) [58]. It was concluded that the voice parameters are scarcely evaluated SFE. However, after FESS, the voice presented transitory changes.

## 3. Conclusions and Future Insights

The present collection of articles added new information and confirmed previous data from different models on sinus floor elevation. It has been also shown that several aspects have still to be clarified, especially about the use of collagen membranes, as well as of graft material in relation to bone formation, osseointegration, volume reduction and damage to the sinus mucosa. Other aspects should be clarified about the surgical treatment of the pseudocysts often occasionally discovered. An accurate evaluation of the CBCT images prior the surgery and after various periods of healing is of fundamental importance.

## References

[1] Pjetursson B.E., Tan W.C., Zwahlen M., Lang N.P. (2008). A systematic review of the success of sinus floor elevation and survival of implants inserted in combination with sinus floor elevation. J. Clin. Periodontol..

[2] Starch-Jensen T., Aludden H., Hallman M., Dahlin C., Christensen A.-E., Mordenfeld A. (2018). A systematic review and meta-analysis of long-term studies (five or more years) assessing maxillary sinus floor augmentation. Int. J. Oral Maxillofac. Surg..

[3] Del Fabbro M., Wallace S.S., Testori T. (2013). Long-term implant survival in the grafted maxillary sinus: A systematic review. Int. J. Periodontics Restor. Dent..

[4] Kawakami S., Botticelli D., Nakajima Y., Sakuma S., Baba S. (2019). Anatomical analyses for maxillary sinus floor augmentation with a lateral approach: A cone beam computed tomography study. Ann. Anat. Anat. Anz..

[5] Lozano-Carrascal N., Salomó-Coll O., Gehrke S.A., Guirado J.L.C., Hernández-Alfaro F., Gargallo-Albiol J. (2017). Radiological evaluation of maxillary sinus anatomy: A cross-sectional study of 300 patients. Ann. Anat. Anat. Anz..

[6] Stacchi C., Andolsek F., Berton F., Perinetti G., Navarra C.O., Di Lenarda R. (2017). Intraoperative Complications During Sinus Floor Elevation with Lateral Approach: A Systematic Review. Int. J. Oral Maxillofac. Implant..

[7] Bornstein M.M., Seiffert C., Maestre-Ferrín L., Fodich I., Jacobs R., Buser D., Von Arx T. (2016). An Analysis of Frequency, Morphology, and Locations of Maxillary Sinus Septa Using Cone Beam Computed Tomography. Int. J. Oral Maxillofac. Implant..

[8] Maestre-Ferrin L., Galan-Gil S., Rubio-Serrano M., Penarrocha-Diago M., Penarrocha-Oltra D. (2010). Maxillary sinus septa: A systematic review. Med. Oral Patol. Oral Cir. Bucal.

[9] Dandekeri S.S., Hegde C., Kavassery P., Sowmya M., Shetty B. (2020). CBCT study of morphologic variations of maxillary sinus septa in relevance to sinus augmentation procedures. Ann. Maxillofac. Surg..

[10] Testori T., Yu S.-H., Tavelli L., Wang H.-L. (2020). Perforation Risk Assessment in Maxillary Sinus Augmentation with Lateral Wall Technique. Int. J. Periodontics Restor. Dent..

[11] Yeung A.W.K., Tanaka R., Khong P.-L., Von Arx T., Bornstein M.M. (2018). Frequency, location, and association with dental pathology of mucous retention cysts in the maxillary sinus. A radiographic study using cone beam computed tomography (CBCT). Clin. Oral Investig..

[12] Kawai T., Tanaka R., Yeung A.W.K., von Arx T., Bornstein M.M. (2018). Frequency and type of incidentally detected radiodensities in the maxillary sinus: A retrospective analysis using cone beam computed tomography (CBCT). Clin. Oral Investig..

[13] Testori T., Rosano G., Taschieri S., Del Fabbro M. (2010). Ligation of an unusually large vessel during maxillary sinus floor augmentation. A case report. Eur. J. oral Implant..

[14] Aimetti M., Romagnoli R., Ricci G., Massei G. (2001). Maxillary sinus elevation: The effect of macrolacerations and microlacerations of the sinus membrane as determined by endoscopy. Int. J. Periodontics Restor. Dent..

[15] Schwartz-Arad D., Herzberg R., Dolev E. (2004). The prevalence of surgical complications of the sinus graft procedure and their impact on implant survival. J. Periodontol..

[16] Khoury F. (1999). Augmentation of the sinus floor with mandibular bone block and simultaneous implantation: A 6-year clinical inves-tigation. Int. J. Oral Maxillofac. Implants.

[17] Lundgren S., Andersson S., Gualini F., Sennerby L. (2004). Bone reformation with sinus membrane elevation: A new surgical technique for maxillary sinus floor augmentation. Clin. Implant Dent. Relat. Res..

[18] Choi B.-H., Zhu S.-J., Jung J.-H., Lee S.-H., Huh J.-Y. (2005). The use of autologous fibrin glue for closing sinus membrane perforations during sinus lifts. Oral Surgery Oral Med. Oral Pathol. Oral Radiol. Endod..

[19] Kawakami S., Lang N.P., Ferri M.A., Alccayhuaman K.A., Botticelli D. (2019). Influence of the Height of the Antrostomy in Sinus Floor Elevation Assessed by Cone Beam Computed Tomography: A Randomized Clinical Trial. Int. J. Oral Maxillofac. Implant..

[20] Kawakami S., Lang N.P., Iida T., Ferri M.A., Alccayhuaman K.A.A., Botticelli D. (2018). Influence of the position of the antrostomy in sinus floor elevation assessed with cone-beam computed tomography: A randomized clinical trial. J. Investig. Clin. Dent..

[21] Pikos M.A. (1999). Maxillary sinus membrane repair: Report of a technique for large perforations. Implant Dent..

[22] Testori T., Wallace S.S., Del Fabbro M., Taschieri S., Trisi P., Capelli M., Weinstein R.L. (2008). Repair of large sinus membrane perforations using stabilized collagen barrier membranes: Surgical techniques with histologic and radiographic evidence of success. Int. J. Periodontics Restor. Dent..

[23] Kim Y.-K., Yun P.-Y., Oh J.-S., Kim S.-G. (2014). Prognosis of closure of large sinus membrane perforations using pedicled buccal fat pads and a resorbable collagen membrane: Case series study. J. Korean Assoc. Oral Maxillofac. Surg..

[24] Scala A., Botticelli D., Rangel I.G., de Oliveira J.A., Okamoto R., Lang N.P. (2010). Early healing after elevation of the maxillary sinus floor applying a lateral access: A histological study in monkeys. Clin. Oral Implant. Res..

[25] Scala A., Botticelli D., Faeda R.S., Rangel-Garcia I., de Oliveira J.A., Lang N.P. (2011). Lack of influence of the Schneiderian membrane in forming new bone apical to implants simultaneously installed with sinus floor elevation: An experimental study in monkeys. Clin. Oral Implant. Res..

[26] Quirynen M., Lefever D., Hellings P., Jacobs R. (2014). Transient swelling of the Schneiderian membrane after transversal sinus augmentation: A pilot study. Clin. Oral Implant. Res..

[27] Nosaka Y., Nosaka H., Arai Y. (2015). Complications of postoperative swelling of the maxillary sinus membrane after sinus floor aug-mentation. J. Oral Sci. Rehabil..

[28] Temmerman A., Van Dessel J., Cortellini S., Jacobs R., Teughels W., Quirynen M. (2017). Volumetric changes of grafted volumes and the Schneiderian membrane after transcrestal and lateral sinus floor elevation procedures: A clinical, pilot study. J. Clin. Periodontol..

[29] Hirota A., Lang N.P., Ferri M., Fortich Mesa N., Apaza Alccayhuaman K.A. (2019). Botticelli Tomographic evaluation of the influence of the placement of a collagen membrane subjacent to the sinus mucosa during maxillary sinus floor augmentation: A randomized clinical trial. Int. J. Implant. Dent..

[30] Imai H., Lang N., Ferri M., Hirota A., Alccayhuaman K. (2020). Tomographic Assessment on the Influence of the Use of a Collagen Membrane on Dimensional Variations to Protect the Antrostomy After Maxillary Sinus Floor Augmentation: A Randomized Clinical Trial. Int. J. Oral Maxillofac. Implant..

[31] Sakuma S., Ferri M., Imai H., Mesa N.F., Victorio D.J.B., Alccayhuaman K.A.A., Botticelli D. (2020). Involvement of the maxillary sinus ostium (MSO) in the edematous processes after sinus floor augmentation: A cone-beam computed tomographic study. Int. J. Implant Dent..

[32] Hsu Y., Rosen P.S., Choksi K., Shih M., Ninneman S., Lee C. (2022). Complications of sinus floor elevation procedure and management strategies: A systematic review. Clin. Implant Dent. Relat. Res..

[33] Urban I.A., Nagursky H., Church C., Lozada J.L. (2012). Incidence, diagnosis, and treatment of sinus graft infection after sinus floor elevation: A clinical study. Int. J. Oral Maxillofac. Implant..

[34] Doud Galli S.K., Lebowitz R.A., Giacchi R.J., Glickman R., Jacobs J.B. (2001). Chronic sinusitis complicating sinus lift surgery. Am. J. Rhinol..

[35] Nolan P.J., Freeman K., Kraut R.A. (2014). Correlation between schneiderian membrane perforation and sinus lift graft outcome: A Retrospective evaluation of 359 augmented sinus. J. Oral Maxillofac. Surg..

[36] Godoy E., Alccayhuaman K.A., Botticelli D., Amaroli A., Balan V., Silva E., Xavier S.P. (2021). Osteoconductivity of Bovine Xenograft Granules of Different Sizes in Sinus Lift: A Histomorphometric Study in Rabbits. Dent. J..

[37] Costa M., Botticelli D., Moses O., Omori Y., Fujiwara S., Silva E., Xavier S. (2021). Maxillary Sinus Augmentation Using Ceramic Alloplastic Granules or Paste: An Experimental Study in Rabbits. Dent. J..

[38] Iida T., Silva E.R., Lang N.P., Alccayhuaman K.A.A., Botticelli D., Xavier S. (2018). Histological and micro-computed tomography evaluations of newly formed bone after maxillary sinus augmentation using a xenograft with similar density and mineral content of bone: An experimental study in rabbits. Clin. Exp. Dent. Res..

[39] Iida T., Baba S., Botticelli D., Masuda K., Xavier S.P. (2020). Comparison of histomorphometry and microCT after sinus augmentation using xenografts of different particle sizes in rabbits. Oral Maxillofac. Surg..

[40] Perini A., Viña-Almunia J., Carda C., de Llano J.J.M., Botticelli D., Peñarrocha-Diago M. (2021). Influence of the Use of a Collagen Membrane Placed on the Bone Window after Sinus Floor Augmentation—An Experimental Study in Rabbits. Dent. J..

[41] Kotsu M., Alccayhuaman K.A.A., Ferri M., Iezzi G., Piattelli A., Mesa N.F., Botticelli D. (2022). Osseointegration at Implants Installed in Composite Bone: A Randomized Clinical Trial on Sinus Floor Elevation. J. Funct. Biomater..

[42] Tanaka K., Iezzi G., Piattelli A., Ferri M., Mesa N.F., Alccayhuaman K.A.A., Botticelli D. (2019). Sinus Floor Elevation and Antrostomy Healing: A Histomorphometric Clinical Study in Humans. Implant Dent..

[43] Favero R., Alccayhuaman K.A.A., Botticelli D., Xavier S.P., Balan V.F., Macchi V., De Caro R. (2021). Sinus Mucosa Thinning and Perforations after Sinus Lifting Performed with Different Xenografts: A Histological Analysis in Rabbits. Dent. J..

[44] Miki M., Botticelli D., Silva E., Xavier S., Baba S. (2021). Incidence of Sinus Mucosa Perforations During Healing After Sinus Elevation Using Deproteinized Bovine Bone Mineral as Grafting Material: A Histologic Evaluation in a Rabbit Model. Int. J. Oral Maxillofac. Implant..

[45] Kato S., Botticelli D., De Santis E., Kanayama M., Ferreira S., Rangel-Garcia I. (2021). Sinus mucosa thinning and perforation after sinus augmentation. A histological study in rabbits. Oral Maxillofac. Surg..

[46] Omori Y., Botticelli D., Ferri M., Delgado-Ruiz R., Balan V.F., Xavier S.P. (2021). Argon Bioactivation of Implants Installed Simultaneously to Maxillary Sinus Lifting without Graft. An Experimental Study in Rabbits. Dent. J..

[47] Canullo L., Tallarico M., Botticelli D., Alccayhuaman K.A.A., Neto E.C.M., Xavier S.P. (2018). Hard and soft tissue changes around implants activated using plasma of argon: A histomorphometric study in dog. Clin. Oral Implant. Res..

[48] Masuda K., Silva E., Alccayhuaman K., Botticelli D., Xavier S.P. (2020). Histologic and Micro-CT Analyses at Implants Placed Immediately After Maxillary Sinus Elevation Using Large or Small Xenograft Granules: An Experimental Study in Rabbits. Int. J. Oral Maxillofac. Implant..

[49] Iida T., Neto E.C.M., Botticelli D., Alccayhuaman K.A.A., Lang N.P., Xavier S.P. (2017). Influence of a collagen membrane positioned subjacent the sinus mucosa following the elevation of the maxillary sinus. A histomorphometric study in rabbits. Clin. Oral Implant. Res..

[50] Aimetti M., Massei G., Morra M., Cardesi E., Romano F. (2008). Correlation between gingival phenotype and Schneiderian membrane thickness. Int. J. Oral Maxillofac. Implant..

[51] Botticelli D., Lang N.P. (2017). Dynamics of osseointegration in various human and animal models—A comparative analysis. Clin. Oral Implant. Res..

[52] Omori Y., Nakajima Y., Imai H., Yonezawa D., Ferri M., Alccayhuaman K.A., Botticelli D. (2021). Influence of Anatomical Parameters on the Dimensions of the Subantral Space and Sinus Mucosa Thickening after Sinus Floor Elevation. A Retrospective Cone Beam Computed Tomography Study. Dent. J..

[53] Morimoto A., Kobayashi N., Ferri M., Iezzi G., Piattelli A., Mesa N.F., Botticelli D. (2022). Influence on Implant Bone Healing of a Collagen Membrane Placed Subjacent the Sinus Mucosa—A Randomized Clinical Trial on Sinus Floor Elevation. Dent. J..

[54] Scala A., Lang N.P., Velez J.U., Favero R., Bengazi F., Botticelli D. (2016). Effects of a collagen membrane positioned between augmentation material and the sinus mucosa in the elevation of the maxillary sinus floor. An experimental study in sheep. Clin. Oral Implant. Res..

[55] Favero V., Lang N.P., Canullo L., Velez J.U., Bengazi F., Botticelli D. (2015). Sinus floor elevation outcomes following perforation of the Schneiderian membrane. An experimental study in sheep. Clin. Oral Implant. Res..

[56] Nosaka Y., Nosaka H., Nakajima Y., Tanioka T., Botticelli D., Baba S. (2021). A Reliable Surgical Procedure for Sinus Floor Augmentation with Antral Pseudocysts. Dent. J..

[57] Kato S., Omori Y., Kanayama M., Hirota A., Ferri M., Alccayhuaman K.A., Botticelli D. (2021). Sinus Mucosa Thickness Changes and Ostium Involvement after Maxillary Sinus Floor Elevation in Sinus with Septa. A Cone Beam Computed Tomography Study. Dent. J..

[58] Delgado-Ruiz R., Botticelli D., Romanos G. (2022). Temporal and Permanent Changes Induced by Maxillary Sinus Lifting with Bone Grafts and Maxillary Functional Endoscopic Sinus Surgery in the Voice Characteristics—Systematic Review. Dent. J..

